# The Predictive Value of Global Longitudinal and Circumferential Strains in Hypertensive Patients: 10-Year Follow-Up

**DOI:** 10.3390/jcm13195799

**Published:** 2024-09-28

**Authors:** Marijana Tadic, Tamara Filipovic, Jelena Suzic, Anka Majstorovic, Biljana Pencic, Vladan Vukomanovic, Cesare Cuspidi, Vera Celic

**Affiliations:** 1 Department of Cardiology, University Clinical Hospital Center “Dr. Dragisa Misovic-Dedinje”, 11000 Belgrade, Serbia; 2 Faculty of Medicine, Institute for Rehabilitation, University of Belgrade, 11000 Belgrade, Serbia; 3 Department of Medicine and Surgery, University of Milano-Bicocca, 20132 Milan, Italy

**Keywords:** hypertension, left ventricle, strain, prediction, MACE

## Abstract

**Background:** The aim of the current study was to investigate the predictive value of a multidirectional LV strain on adverse outcomes in a large population of uncomplicated hypertensive patients who were followed for a mean period of 10 years. **Methods:** This retrospective study included 591 recently diagnosed hypertensive patients who underwent clinically indicated echocardiography between January 2010 and December 2014 and were followed for a mean period of 10 years. Global longitudinal, circumferential and radial strains (GLS, GCS and GRS) were measured by 2D speckle tracking imaging. The primary outcome was a MACE occurrence defined by all-cause mortality, cardiovascular mortality, myocardial infarction, coronary artery by-pass, coronary stent implantation, stroke, development of heart failure and the occurrence of atrial fibrillation during follow-up. **Results:** Our results showed that GLS, GCS and GRS were significantly lower in patients who experienced MACE. Age, male gender, systolic blood pressure, left ventricular hypertrophy (LVH) and left atrial enlargement (LAE) were associated with MACE occurrence. Reduced GLS [OR 1.15; 95%CI: 1.01–1.30] and reduced GCS [OR 1.1; 95%CI: 1.02–1.22] were related with MACE independently of clinical characteristics, LV systolic and diastolic function, as well as LVH. Reduced GRS was not independently associated with adverse outcomes. **Conclusions:** Reduced GLS and GCS were independently associated with adverse outcomes during 10-year follow-up in patients who were recently diagnosed and uncomplicated hypertensive patients at the baseline.

## 1. Introduction

The evaluation of the left ventricular global longitudinal strain (LV GLS) was introduced almost two decades ago, and a large burden of evidence proved its advantage over traditional parameters of LV systolic function, such as ejection fraction (EF), in predicting adverse outcomes in a wide spectrum of cardiovascular diseases [1,2,3]. The development of artificial intelligence and the implementation of robust software incorporated into modern echocardiographic machines have enabled swift and convenient GLS analysis, which has made it possible for GLS determination in everyday clinical practice. Guidelines also support this evaluation and inclusion of GLS in routine echocardiographic reports [4,5]. However, despite the evidence, recommendations and technique developments, GLS has not been routinely used in everyday practice in most echocardiographic laboratories.

The evaluation of GLS and global circumferential strain (GLS) showed significant reduction in patients with prehypertension, newly diagnosed, treated, uncontrolled or resistant hypertension despite normal LVEF [6,7]. Myocardial work is a novel set of parameters derived from GLS and systolic blood pressure (SBP), which provides even more insightful information about LV mechanics [8]. It includes SBP as the major confounding factor for GLS assessment, and therefore, represents an even more robust parameter that remains to show its predictive value in a hypertensive population in the future.

Data on GCS are not very consistent, as this parameter has not been extensively evaluated. Some investigations reported GCS reduction in hypertensive patients [9], while others did not show significant difference in GCS between hypertensive and normotensive subjects [10]. A study that conducted a 4-year follow-up of uncomplicated hypertensive patients revealed the predictive importance of GLS, independently of LV hypertrophy (LVH) and LV diastolic function parameters [10]. These were the reasons why consensus for echocardiographic evaluation in hypertensive patients suggested the GLS evaluation [5]. The importance of GLS during longer follow-up in hypertensive populations and, particularly, the predictive value of reduced GCS and GRS have not been investigated so far.

Non-invasive CV imaging and, primarily, echocardiography significantly improved our role in the prevention and detection of subclinical changes that have significant predictive importance in patients with various risk factors, including arterial hypertension. However, the importance of other modalities, such as Doppler imaging, cardiac magnetic resonance, and cardiac CT and multimodality approaches should be strongly supported in patients with CV risk factors in order to timely detect these impairments [11].

The aim of the current study was to investigate the predictive value of multidirectional LV strain (GLS, GCS and GRS) in a large population of uncomplicated hypertensive patients who were followed-up for a mean period of 10 years. The primary outcome examined in this study was MACE (major cardiovascular events) that were defined as the composite endpoint which consists of all-cause mortality, cardiovascular mortality, myocardial infarction, coronary artery by-pass, coronary stent implantation, stroke, development of heart failure and the occurrence of atrial fibrillation.

## 2. Materials and Methods

This retrospective study included 591 recently diagnosed hypertensive patients who underwent clinically indicated echocardiography between January 2010 and December 2014. Participants with heart failure, coronary artery disease, previous stroke, atrial fibrillation, congenital heart disease, valvular heart disease, kidney failure, excessive obesity, type 2 diabetes mellitus or neoplastic disease were excluded from the study. Anthropometric measures and laboratory analyses were taken from all the subjects included in the study. Body mass index (BMI) was calculated for each patient.

Clinic arterial BP values were obtained in the morning hours by measuring the average value of the two consecutive measurements in the sitting position taken 5 min apart. BP was obtained in at least two separate occasions.

Clinical parameters (medical history and comorbidities, medication, and laboratory analyses) were collected by an investigator blinded to the echocardiographic findings at the time of the echocardiographic examination. This study was approved by the local Ethics Committee, and informed consent was obtained from all the participants.

### 2.1. Echocardiography

Echocardiographic examination was performed by a Vivid 7 ultrasound machine (GE Healthcare, Horten, Norway). The values of all 2D parameters were calculated as the average value of three consecutive cardiac cycles. LV diameters, interventricular septum and relative wall thickness were determined according to the guidelines [4]. LVEF was assessed by the biplane method. LV mass was calculated using the formula of the American Society of Echocardiography [4] and indexed for body surface area. LV hypertrophy (LVH) was defined as LVMI ≥ 95 g/m^2^ in women and ≥ 115 g/m^2^ for men. Left atrial (LA) volume was measured by the biplane method in 4- and 2-chamber views and indexed for BSA. Left atrial enlargement (LAE) was defined as LAVI ≥ 34 mL/m^2^ for both genders. Transmitral Doppler inflow and tissue Doppler velocities were obtained according to the guidelines [12].

### 2.2. Two-Dimensional LV Strain Analysis

Two-dimensional speckle tracking imaging was assessed using three consecutive cardiac cycles [4], and a commercially available software Q-analysis (EchoPAC 201, GE-Healthcare, Horten, Norway) was used for 2D strain analysis. The 2D speckle tracking analysis was performed in three apical (4- and 2-chamber, long-axis) views and a parasternal short-axis view at the papillary muscle level. Apical views were used for the assessment of longitudinal and circumferential strains, and short-axis parasternal view was used for the evaluation of circumferential and radial strains.

A modified 2D strain software (Q-analysis) was used for the evaluation of multilayer longitudinal and circumferential strains. GLS was evaluated in an apical 4-chamber, 2-chamber and apical long-axis view, whereas GCS and GRS were calculated in short-axis at the level of papillary muscles. The automatic tracking of the endocardial contour was assessed in the end-systole. The software automatically provided LV myocardium tracking and the region of interest was manually corrected to confirm the optimal tracking of the endocardium and epicardium in order to include the entire LV thickness in all observed echocardiographic views. After outlining the region of interest, the software divided the LV into 6 segments in the 4-chamber, 2-chamber, apical long-axis view and assessed GLS as the average of GLS in 3 three views.

### 2.3. Outcome

The primary outcome was MACE occurrence (all-cause mortality, cardiovascular mortality, myocardial infarction, coronary artery by-pass, coronary stent implantation, stroke, development of heart failure, occurrence of atrial fibrillation) during follow-up. The study protocol required follow-up of all patients for a mean period of 10 years after initial evaluation. All data during follow-up were obtained during the clinical visit, via telephone communication or via the referring physician. During follow-up, 46 patients were not reached, which is why they were excluded from this study and further analysis.

### 2.4. Statistical Analysis

Continuous variables were presented as mean ± standard deviation showing normal distribution and they were compared using the t-test for two independent samples. Differences in proportions were compared by the χ^2^ test. Univariate and multivariate logistic regression analyses were used for the determination of the association between clinical parameters, including reduced LV longitudinal and circumferential strain, and MACE occurrence. Considering the fact that the accurate date of adverse event occurrence was not detectable in all patients, Kaplan–Meier and Cox regression analyses were not used. Cut-off values for normal global longitudinal, circumferential and radial strains were −20%, −21% and 35%, respectively. Model 1 included univariate and multivariate logistic regression analyses with GLS as the dependent variable, whereas Model 2 analyzed GCS as the dependent variable and Model 3 evaluated GRS as the dependent variable. Intra- and inter-observer variability for LV strain parameters was assessed in 25 study patients by the evaluation of the intra-class correlation coefficients (ICCs). The *p*-value < 0.05 was considered statistically significant.

## 3. Results

Patients who experienced MACE (176 out of 591, 29.7%) were significantly older, predominantly males and more obese than those who did not experience MACE (Table 1). There was no difference in systolic and diastolic BP, glucose, creatinine, cholesterol and triglyceride levels between groups (Table 1). The medication history showed no difference in the prevalence of taking different antihypertensive medications and statins at the baseline (ACEI/ARB, beta blockers, calcium channels blockers and diuretics) between the two observed groups (Table 1).

### 3.1. Conventional Echocardiographic Measurements

LV end-diastolic diameter, LV interventricular thickness and relative wall thickness were higher among patients with MACE (Table 2). LV mass index and LA volume index were higher in patients who experienced MACE than in their counterparts (Table 2). LVEF was lower in MACE group of patients. Mitral E/A rand E/e′ ratios were lower in MACE patients (Table 2).

**Figure 1:**
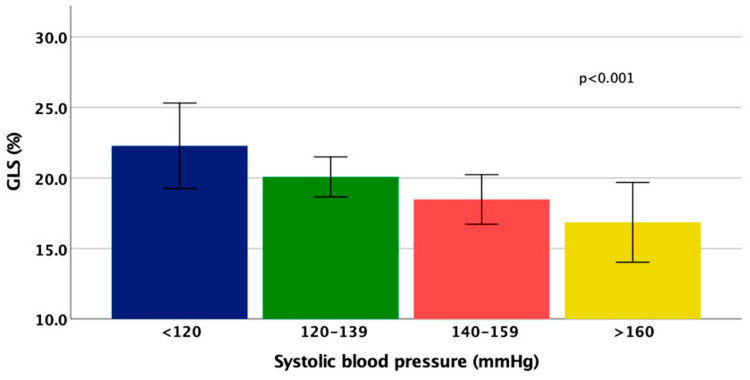
GLS in patients with different systolic blood pressure.

Two-dimensional LV global longitudinal, circumferential and radial strains were significantly lower in patients who experienced MACE than in those who did not (Table 2). Figure 1 illustrates significant and gradual GLS reduction with elevation of SBP.

### 3.2. Logistic Regression Analyses

**Figure 2:**
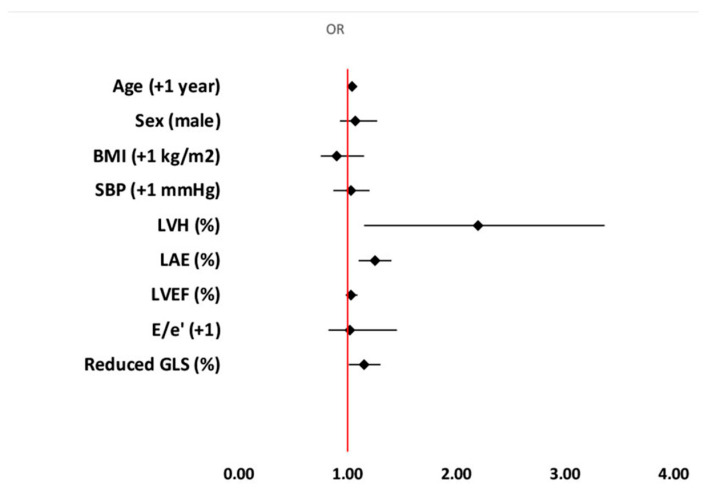
Parameters related with MACE during 10-year follow-up involving GLS as independent predictor. Abbreviations: BMI—body mass index; E—early diastolic mitral flow (pulse Doppler); e′—the average between early diastolic flow velocity across the septal and lateral segments of mitral (e′) annulus (tissue Doppler); GLS—global longitudinal strain; LAE—left atrial enlargement; LVH—left ventricular hypertrophy; LVEF—left ventricular ejection fraction.

**Figure 3:**
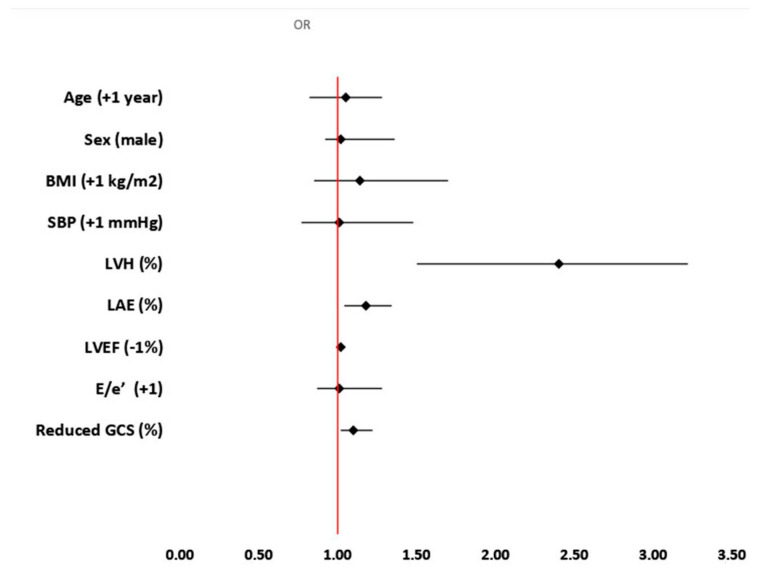
Parameters related with MACE during 10-year follow-up including GCS as independent predictor. Abbreviations: BMI—body mass index; E—early diastolic mitral flow (pulse Doppler); e′—the average between early diastolic flow velocity across the septal and lateral segments of mitral (e′) annulus (tissue Doppler); GLS—global longitudinal strain; LAE—left atrial enlargement; LVH—left ventricular hypertrophy; LVEF—left ventricular ejection fraction.

Univariate logistic regression analysis showed that age, male gender and systolic blood pressure of clinical characteristics were associated with MACE occurrence (Table 3). Echocardiographic parameter of LV structure (LVH), but not index of LV diastolic function (E/e′), was associated with MACE occurrence. Left atrial enlargement was also related with MACE (Table 3). Multivariable logistic regression analysis showed that only age [OR 1.04; 95%CI: 1.01–1.08], LVH [OR 2.2; 95%CI: 1.15–3.36], LAE [OR 1.25; 95%CI: 1.10–1.40] and reduced GLS [OR 1.15; 95%CI: 1.01–1.30] were independently associated with the primary outcome (Table 3). Model 2, which consisted of the same parameters excluding only GCS, showed that LVH [OR 2.40; 95%CI: 1.50–3.32], LAE [OR 1.18; 95%CI: 1.04–1.34] and reduced GCS [OR 1.1; 95%CI: 1.02–1.22] were independently related with adverse outcome reflected by MACE (Table 3). Model 3 investigated the predictive value of GRS among the same set of variables as in Models 1 and 2 (except GLS and GCS) and showed that only systolic BP [OR 1.03; 95%CI: 1.01–1.05] and LVH [OR 1.65; 95%CI: 1.12–2.17] were independent predictors of MACE in hypertensive patients followed for 10 years (Table 3). Figure 2 and Figure 3 represent results of multivariable regression analyses for GLS and GCS.

### 3.3. Intra- and Inter-Observer Variability

Inter- and intra-observer variability is low for all parameters of 2D LV GLS, GCS and GRS, as provided in Table 4.

## 4. Discussion

The current study provided several important findings that may encourage future routine echocardiographic evaluation of LV mechanics in hypertensive patients. Our findings revealed that reduced GLS and GCS were predictors of MACE independently of age, BMI, LVH, LV systolic and diastolic function during a period of 10 years.

An increase in LV afterload and interstitial fibrosis are characteristic of arterial hypertension and hypertensive heart disease, and both are associated with reduced LV longitudinal function, as assessed by GLS [13,14,15], and this is detectable not only in patients with LVH, but also in those with normal LV geometry and LVEF [8]. Multidirectional LV strain is related with parameters of LV diastolic function and LV filling pressure (E/A, E/e′) [14]. Therefore, GLS represents an important marker to reveal subclinical impairment in both LV contraction and relaxation.

The importance of GLS in a hypertensive population has been previously described and our study group demonstrated subclinical subtle results in patients with various types of hypertension, including prehypertension, newly diagnosed and resistant hypertension [6,7], which is much more difficult to prove than cardiac damage in long-standing, uncontrolled and resistant hypertension that are already characterized by advanced LV diastolic dysfunction and LVH. The question that arises is the clinical value of the early detection of reduced GLS. In the absence of large follow-up clinical studies that confirm the clinical importance of GLS in a hypertensive population, it is very difficult to persuade clinicians to put additional effort into LV strain evaluation in these patients. The present data showed that reduced GLS was independent of age, BMI, blood pressure, LV hypertrophy and LV systolic and diastolic dysfunction associated with MACE during 10-year follow-up. This is an important finding because LVH and LV diastolic function, as well as LAE, are frequently diagnosed in hypertensive patients, but our data show that decreased GLS is more important than LV systolic and diastolic function and equally important as LVH in the prognosis of adverse events in uncomplicated hypertensive patients. Interestingly, blood pressure was not independently related with MACE during 10-year follow-up. Saito et al. reported that GLS was related with MACE, independent of clinical parameters and concentric LVH, in 388 hypertensive patients and abnormal LV geometry during 4-year follow-up [10]. Lee et al. followed 95 hypertensive patients for 7 years and revealed no difference in GLS between patients who experienced cardiovascular events and those who did not [15]. The authors found a significant difference in LV layer-specific longitudinal strain, particularly epicardial strain, which represented the only independent predictor of cardiovascular mortality and hospitalization due to heart failure in this population [15].

Data on GCS in a hypertensive population are scarce. Our data previously showed that GCS was reduced in uncontrolled [7] but not in prehypertension hypertension [6]. Published data also showed a significant relationship between BP and GCS. However, Saito et al. did not show that GCS was associated with MACE and suggested that GCS is relatively preserved in comparison with LV longitudinal function [8]. The authors suggested that GCS might not correctly reflect LV mid-wall shortening due to endocardial border tracking. The present findings reveal for the first time the independent association between GCS and MACE, which is of a great clinical importance and supports a more detailed echocardiographic assessment of these patients, including GCS.

Radial myocardial thickening is the best reflection of LV systolic function and even if longitudinal LV function is deteriorated, radial function is still preserved. However, GRS is technically limited with significantly lower reproducibility than GLS and GCS, which is why many studies did not find any deterioration of GRS in hypertensive patients [6,7]. Our previous studies revealed a trend of decreased GRS in hypertensive patients, but without reaching statistical significance [6,7]. The current data showed that reduced GRS was associated with MACE, but not independently of LVH and other clinical parameters, in our hypertensive patients. This shows that GRS is important, but not more relevant than LVH and LVEF, for hypertensive patients.

The most important clinical implication of this study arises from the finding that both GLS and GCS were independently associated with adverse cardiovascular outcomes, which not only supports the recommendation to determine GLS during conventional echocardiographic examination, but also to try to expand this to GCS. This is not currently feasible in most echocardiographic laboratories because GCS has not been incorporated in a majority of echocardiographic machine software yet.

There are several limitations of the current study: (i) we included uncomplicated hypertensive patients at baseline, which somewhat limits the generalization of our findings, which particularly refers to the elderly patients with long-lasting hypertension, and hypertensive patients with concomitant comorbidities such as diabetes and ischemic heart disease; (ii) the absence of a normotensive group is an additional limitation because there was no possibility to compare hypertensive patients with normotensive subjects; and (iii) the lack of recommendations regarding threshold values for normal GLS and, moreover, for GCS and GRS.

## 5. Conclusions

LV multidirectional mechanics is impaired in hypertensive patients. Not only did our study reveal that GLS, GCS and GRS were significantly decreased in a large number of uncomplicated hypertensive patients, but it also reported that all three parameters were associated with adverse cardiovascular outcomes during 10-year follow-up. However, only LV GLS and GCS were related with MACE independently of clinical and echocardiographic parameters. Our findings underlined the importance of GLA and GCS routine assessments in everyday clinical practice due to their predictive value in hypertensive patients. The current data support guidelines and emphasize the importance of GCS evaluation in hypertensive patients and provide a solid foundation for future recommendations for the echocardiographic assessment of hypertensive patients.

## Figures and Tables

**Table 1:** Demographic characteristics and clinical parameters of study population.

	All Patients (n = 591)	MACE (n = 176)	Non-MACE (n = 415)	*p*
Age (years)	54 ± 11	56 ± 10	53 ± 11	0.002
Male (%)	311 (53)	74 (59)	237 (51)	<0.001
BMI (kg/m^2^)	26.8 ± 3.0	27.4 ± 3.1	26.6 ± 2.9	0.003
Systolic blood pressure (mmHg)	145 ± 10	146 ± 11	145 ± 10	0.281
Diastolic blood pressure (mmHg)	93 ± 9	92 ± 8	93 ± 9	0.203
Plasma glucose (mmol/L)	5.3 ± 1.3	5.4 ± 1.4	5.2 ± 1.3	0.200
Total cholesterol (mmol/L)	5.9 ± 2	6.0 ± 1.9	5.8 ± 2.0	0.260
Triglycerides (mmol/L)	2.0 ± 0.3	2.1 ± 0.9	2.0 ± 0.8	0.182
Serum creatinine (mmol/L)	94 ± 21	93 ± 20	95 ± 21	0.283
ACEI/ARBs (%)	375 (63)	114 (65)	261 (63)	0.709
Beta blockers (%)	153 (26)	49 (28)	104 (25)	0.474
Calcium channel blockers (%)	253 (43)	79 (45)	174 (42)	0.525
Diuretics (%)	171 (29)	55 (31)	116 (28)	0.429
Statins (%)	156 (26)	44 (25)	112 (27)	0.683

ACEI—angiotensin-converting enzyme inhibitors; ARB—angiotensin II receptor blockers; BMI—body mass index.

**Table 2:** Echocardiographic parameters of left ventricular structure and function in study population.

	All Patients (n = 591)	MACE (n = 176)	Non-MACE (n = 415)	*p*
LVEDD (mm)	47.9 ± 4.1	48.9 ± 4.2	47.5 ± 4.1	<0.001
IVS (mm)	10 ± 1	10.4 ± 1.1	9.8 ± 1.0	<0.001
RWT	0.42 ± 0.11	0.43 ± 0.12	0.41 ± 0.10	0.037
LAVI (mL/m^2^)	30.6 ± 4.1	31.5 ± 4.0	30.2 ± 4.2	<0.001
LVMI (g/m^2^)	89.1 ± 10.8	95.8 ± 11.9	86.3 ± 10.3	<0.001
EF (%)	63 ± 4	62 ± 4	63 ± 4	0.006
E/A ratio	0.91 ± 0.21	0.82 ± 0.24	0.94 ± 0.18	<0.001
E/e′	9.2 ± 2.7	10.3 ± 2.5	8.7 ± 2.8	<0.001
Two-dimensional mechanical parameters				
Global strain (%)				
Longitudinal	−19.3 ± 2.4	−18.1 ± 2.1	−19.8 ± 2.5	<0.001
Circumferential	−21.8 ± 2.7	−20.8 ± 2.5	−22.3 ± 2.8	<0.001
Radial	39 ± 9.2	36.5 ± 8.4	40.1 ± 9.6	<0.001

A—Late diastolic mitral flow (pulse Doppler); E—early diastolic mitral flow (pulse Doppler); e′—the average between early diastolic flow velocity across the septal and lateral segments of mitral (e′) annulus (tissue Doppler); EF—ejection fraction; IVS—interventricular septum; LAVI—left atrial volume index; LVEDD—left ventricle end-diastolic dimension; LVMI—left ventricular mass index; RWT—relative wall thickness.

**Table 3:** Parameters related with MACE during 10-year follow-up.

	Predictors of MACE											
	Univariate Analysis			Multivariate Analysis			Multivariate Analysis			Multivariate Analysis		
	OR	95% CI	*p*	OR	95% CI	*p*	OR	95% CI	*p*	OR	95% CI	*p*
	Model 1 (Reduced GLS)						Model 2 (Reduced GCS)			Model 3 (Reduced GRS)		
Age (+1 year)	1.08	1.02–1.14	0.004	1.04	1.01–1.08	0.038	1.05	0.82–1.28	0.150	1.07	0.92–1.21	0.165
Sex (male)	1.10	1.02–1.19	0.037	1.07	0.93–1.27	0.205	1.02	0.92–1.36	0.206	1.12	0.80–1.78	0.210
BMI (+1 kg/m^2^)	1.04	0.93–1.24	0.094	0.90	0.75–1.15	0.220	1.14	0.85–1.70	0.180	1.21	0.85–1.49	0.098
SBP (+1 mmHg)	1.06	1.01–1.14	0.002	1.03	0.87–1.20	0.093	1.01	0.77–1.48	0.135	1.03	1.01–1.05	0.011
LVH (%)	2.85	1.40–4.25	<0.001	2.20	1.15–3.36	<0.001	2.40	1.50–3.32	<0.001	1.65	1.12–2.17	0.026
LAE (%)	1.40	1.07–1.78	<0.001	1.25	1.10–1.40	0.025	1.18	1.04–1.34	0.047	1.09	0.95–1.31	0.139
LVEF (+1%)	1.06	1.01–1.11	0.004	1.03	0.98–1.09	0.058	1.02	0.99–1.05	0.062	1.04	0.93–1.26	0.214
E/e′	1.09	0.87–1.36	0.182	1.02	0.82–1.45	0.127	1.01	0.87–1.28	0.249	1.03	0.83–1.29	0.240
Reduced GLS (%)	1.26	1.04–1.60	<0.001	1.15	1.01–1.30	0.011		-			-	
Reduced GCS (%)	1.16	1.03–1.40	0.005		-		1.10	1.02–1.22	0.041		-	
Reduced GRS (%)	1.07	1.02–1.12	0.009		-			-		1.06	0.91–1.23	0.132

BMI—Body mass index; E—early diastolic mitral flow (pulse Doppler); e′—the average between early diastolic flow velocity across the septal and lateral segments of mitral (e′) annulus (tissue Doppler); LAE—left atrial enlargement; LVEF—left ventricular ejection fraction; LVH—left ventricular hypertrophy; SBP—systolic blood pressure. Model 1 included all listed parameters and used medications, but does not include GCS and GRS; Model 2 does not include GLS and GRS; and Model 3 does not include GLS and GCS.

**Table 4:** Inter- and intra-observer variability.

	ICC	95% CI	*p*
Inter–observer variability			
Global longitudinal strain	0.918	0.901–0.940	<0.001
Global circumferential strain	0.904	0.882–0.932	<0.001
Global radial strain	0.825	0.758–0.941	<0.001
Intra–observer variability			
Global longitudinal strain	0.938	0.907–0.972	<0.001
Global circumferential strain	0.920	0.890–0.957	<0.001
Global radial strain	0.845	0.770–0.924	<0.001

CI—Confidential interval; ICC—inter-class correlation.

## Data Availability

Data are not available online and may be available at reasonable request.
